# Chronic pain and post-traumatic stress in older patients with psychiatric disorders during the Covid-19 pandemic: co-occurrence and influence of attachment and personality factors

**DOI:** 10.1192/j.eurpsy.2026.11479

**Published:** 2026-07-31

**Authors:** H. Saint-Martin, J.-M. Dorey, M. Herrmann, B. Laurent, C. Lebrun-Givois, C. Perrot, A. Edjolo, L. Ouss, E. Pongan, I. Rouch

**Affiliations:** 1 Department of Aging Psychiatry, Le Vinatier Hospital, Bron; 2 Neuropain Team, Lyon Neuroscience Research Center, Lyon; 3 Memory Clinical and Research Center, University Hospital of Saint-Etienne , Saint-Etienne; 4 Department of Child and Adolescent Psychiatry, APHP, Necker Hospital, Paris; 5 ACTIVE Team, Bordeaux Population Health Center, University of Bordeaux, Bordeaux, France

## Abstract

**Introduction:**

The **Covid-19 pandemic** context may have had numerous **effects on the health of older patients with psychiatric disorders** (PD), confronting them with a new source of stress and hindering their access to care. These effects could have included the onset of **post-traumatic stress** (PTSD) symptoms — as well as **chronic pain** (CP), which often occurs alongside trauma.

CP and PTSD share a **common pathological mechanism** and numerous studies have highlighted the **frequent co-occurrence** of CP and PTSD.

**Explanatory models** for this link **still lack evidence**. Furthermore, they have **not been tested in older and/or PD populations**.

**Objectives:**

The aim of this study was to assess the **long-term effects of the pandemic on both CP and PTSD**; the **comorbidity** of the two disorders; and to identify **common psychological risk factors.**

**Methods:**

**Design:** Medical interviews were conducted during and after (12 and 18 months later) the first lockdown.

**Setting:** The STERACOVID longitudinal cohort study, conducted in two French hospitals.

**Participants:** 71 patients aged 65 or over; treated in an outpatient psychiatric service; and free of major neurocognitive disorders.

**Measurements:** Validated scales were used to assess CP (ICD-11 criteria); PTSD (PCL-S); personality traits (BFI-Fr); attachment style (RSQ); and coping strategies (BRIEF-COPE). χ² and Student’s t-tests, analyses of variance and logistic regression were used to compare patients with or without CP and/or PTSD, in terms of attachment styles, personality traits and coping strategies.

**Results:**

**Prevalence** of :

**CP** : 17% at T1 to 55% at T2, p = .005*

**PTSD** : 31% at T2

**Association** between CP and PTSD : PTSD **frequency** : 41% of CP2+ patients vs. 19% of CP2- patients, p = .043*

**Attachment style:** CP1-CP2+ more **fearful** than CP1-CP2- (OR = 2.71, p = 0.046*)

PTSD2+ more **fearful** (3.40 vs. 2.90, F = 2.43, p = 0.020*) and **preoccupied** (2.53 vs. 2.12, F = 5.66, p = 0.026*) than PTSD2-

CP2+PTSD2+ and CP2+PTSD2- more **fearful** than CP2-PTSD2-

**Personality factors:** CP1+ more **extraverted** than CP1-CP2- (OR = 3.61, p = 0.050*)

PTSD2+ more **neurotic** than PTSD2- (3.81 vs. 3.31, F = 6.35, p = 0.014*)

CP2+PTSD2- more **extraverted** than CP-PTSD2-

**Coping strategies:** no associations

**Conclusions:**

CP and PTSD were **frequent** and **often co-occurring**, 18 months after the first lockdown. Our study identified factors associated with a higher risk of developing CP and/or PTSD in the pandemic context :

**Attachment** styles: **insecure-anxious profiles** were more likely to develop CP and/or PTSD

**Personality** traits: **extraversion** predicted CP, **neuroticism** predicted PTSD but not CP

These results are in line with the **anxiety sensitivity** hypothesis: common vulnerability etiology for CP and PTSD, predicted by neuroticism and extraversion traits, and insecure-anxious attachment styles.

**Disclosure of Interest:**

None Declared

